# Decoupled Interface Passivation Enabled by Directional Ion Migration in Lithium‐Ion Batteries

**DOI:** 10.1002/smll.74978

**Published:** 2026-08-02

**Authors:** Juhwi Park, Jooeun Byun, Chae Rim Lee, Hyun‐seung Kim

**Affiliations:** ^1^ Advanced Batteries Research Center Korea Electronics Technology Institute 25, Saenari‐ro Seongnam Republic of Korea; ^2^ Department of Battery Science and Engineering Sungkyunkwan University Suwon Republic of Korea; ^3^ Department of Energy Sungkyunkwan University Suwon Republic of Korea; ^4^ Department of Future Energy Engineering Sungkyunkwan University Suwon Republic of Korea

**Keywords:** additives, closed‐loop, electrolytes, lithium‐ion batteries, solid electrolyte interphase

## Abstract

Long‐term cycleability of lithium‐ion batteries is limited by interfacial degradation and irreversible lithium loss from parasitic electrolyte decomposition after solid electrolyte interphase formation. In this study, an ionic molecule with individually functionalized cation and anion components is designed to enable the decoupling of interfacial passivation in graphite/NCM811 cells based on directional ion migration. The additive consists of a cation and anion bearing complementary film‐forming functionalities; these two components migrate under electrochemical polarization and selectively decompose at opposite electrodes, leading to the formation of a nitrogen‐rich interphase on the negative electrode and a sulfur‐containing film on the positive electrode. This enables simultaneous stabilization of both interfaces using a single additive. The resulting decoupled passivation suppresses electrolyte decomposition, salt degradation, and continuous film growth, thereby reducing irreversible lithium consumption and impedance growth. Evaluation of 1.2 A h pouch cells demonstrates improved capacity retention, higher Coulombic efficiency, and suppressed resistance growth over 600 cycles at elevated temperatures for the additive‐added cells. This study establishes directional ion migration as a design principle for electrolyte additives and offers a unified strategy for interfacial stabilization in high‐energy‐density lithium‐ion batteries.

## Graphical abstract

An additive with individually functionalized cation and anion components bearing complementary film‐forming functionalities is designed for incorporation in lithium‐ion battery electrolytes. The cations and anions migrate under electrochemical polarization and selectively decompose at opposite electrodes, The resulting decoupled passivation suppresses electrolyte decomposition, salt degradation, and continuous film growth, thereby reducing irreversible lithium consumption and impedance growth.

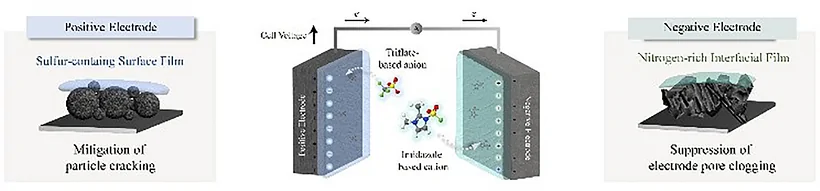

## 1 Introduction

Lithium‐ion batteries (LIBs) play a central role in portable electronics and large‐scale energy‐storage systems, making long‐term cycling stability a critical requirement for practical applications [1, 2, 3, 4, 5, 6, 7, 8, 9, 10, 11, 12]. Because the practical capacity of LIB systems is generally determined based on end‐of‐life specifications, an improvement in the cycling stability also reduces the initially required cell capacity and associated material consumption. Therefore, enhanced cycle life not only improves battery durability but also lowers the manufacturing cost through reduced oversizing margins and lower material consumption during cell fabrication.

Typically, graphite‐based negative electrodes operate at potentials below the electrochemical stability windows of conventional carbonate electrolytes [13, 14, 15, 16, 17, 18]. Consequently, electrolyte decomposition occurs during the initial lithiation process, leading to the formation of a solid electrolyte interphase (SEI) on the surface of the negative electrode [8, 19, 20, 21, 22, 23, 24] while passivation layers are simultaneously formed on the surface of the positive electrode [7, 25, 26, 27, 28, 29, 30]. These interphases then suppress continuous electrolyte decomposition and mitigate Li‐ion consumption during long‐term cycling. However, passivation films formed solely by solvent decomposition often cannot completely prevent parasitic reactions at electrode‐electrolyte interfaces. Therefore, electrolyte additives capable of forming robust and functional passivation films must be used to improve interfacial stability [9, 31, 32, 33].

Conventional electrolyte additives are generally designed to stabilize either the negative or positive electrode interfaces [33, 34, 35, 36, 37]. Consequently, to stabilize both electrode surfaces, the electrolyte is often modified through simultaneous addition of multiple additives, leading to increased electrolyte viscosity and cell cost. Furthermore, recent studies have demonstrated that simultaneous incorporation of multiple additives can alter the additive‐decomposition pathways, thereby diminishing the intrinsic functionality of each additive [38, 39]. To overcome this limitation, multifunctional additives designed to stabilize both electrode interfaces have been proposed [4, 40, 41, 42, 43]. However, because their decomposition is nondirectional with the additive decomposing in similar manner at both electrodes, the interfacial passivation effects of these additives remain difficult to control.

To address this limitation, spontaneous migration of dissociated ions under electrochemical polarization can be exploited to decouple the interfacial passivation reactions by using an ionic additive specifically functionalized for the two different surfaces. During the SEI formation process, cations and anions migrate toward the negative and positive electrodes, respectively. Accordingly, incorporation of an SEI‐forming moiety into the cation component of the additive will selectively stabilize the graphite–electrolyte interface, whereas the introduction of a surface‐film‐forming functionality into the anion component of the additive will selectively stabilize the positive‐electrode interface. Thus, such directional‐ion‐migration‐based design enables selective and efficient construction of interphases on each electrode surface.

In this study, 1‐(fluorosulfonyl)‐2,3‐dimethyl‐1*H*‐imidazol‐3‐ium trifluoromethanesulfonate (FDI‐TMS) was used to validate this strategy. The nitrogen‐containing film‐forming functionality incorporated into the cation (FDI) promotes stabilization of the graphite–electrolyte interface while sulfonate‐based anions (TMS) form a robust interphase on high‐Ni positive electrodes, thereby suppressing interfacial side reactions during cycling. The results show that this ionic additive effectively stabilizes the electrode–electrolyte interfaces and significantly improves the cycleability.

## 2 Results and Discussion

A fundamental analysis of the intrinsic mechanism of the ionic additive and its role in the interfacial film formation was carried out (Figure 1). Figure 1a schematically illustrates how directional ion migration during the SEI formation process enables decoupled passivation of electrode interfaces. In the designed FDI‐TMS molecule, the cation (FDI) contains nitrogen‐based functionalities known to form stable interphases on graphite‐based negative electrodes, whereas the sulfonate‐based moieties of the anion (TMS) can effectively stabilize positive electrodes. During the initial SEI formation process, as the negative electrode potential decreases and the positive electrode potential increases, the electrochemical potential gradient drives the migration of the dissociated cations and anions toward the negative and positive electrodes, respectively. This spontaneous directional migration enables the formation of a nitrogen‐rich SEI on the negative electrode and of a sulfur‐containing surface film on the positive electrode. Accordingly, this ionic additive design allowed the decoupling of interfacial passivation, enabling effective film formation on both electrodes using a single additive.

**FIGURE 1:**
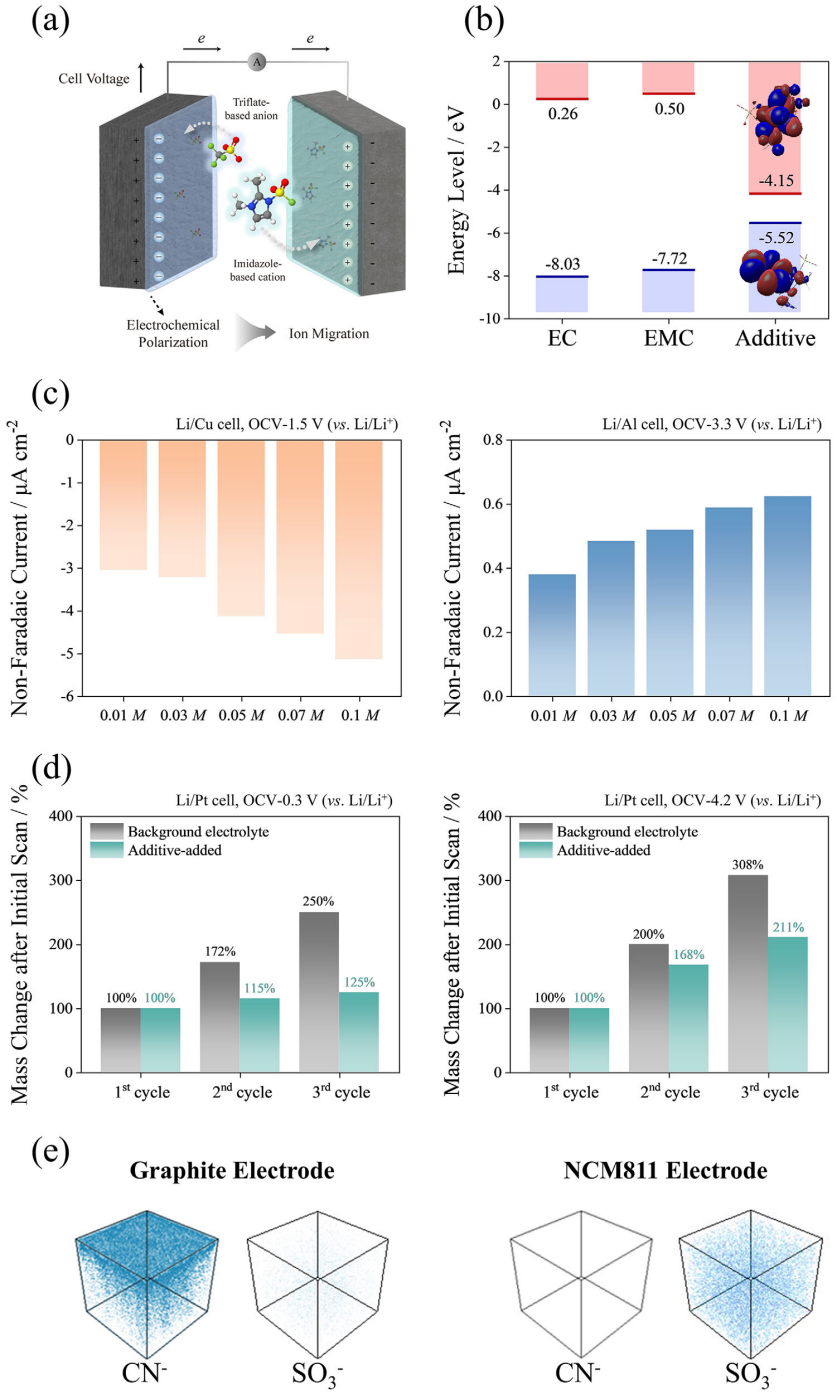
(a) Schematic illustration of the mechanism of decoupled interface passivation induced by directional ion migration, (b) frontier‐orbital energy levels of incorporated solvent molecules and additive, (c) additive‐concentration‐dependent measured double layer charging currents at negative and positive voltage regions, (d) mass change after the initial scan evaluated by EQCM measurements, (e) 3D‐ToF‐SIMS maps for graphite and NCM811 electrodes after formation.

Figure 1b shows the frontier molecular‐orbital energy levels of ethylene carbonate (EC), ethyl methyl carbonate (EMC), and the designed additive. Compared with EC and EMC, the additive exhibited a higher highest occupied molecular orbital (HOMO) level and a lower lowest unoccupied molecular orbital (LUMO) level, suggesting that it can more readily participate in interfacial reactions. This indicated that the additive can act as a more effective film‐forming species than conventional carbonate solvents. The frontier orbital energy level calculations were performed for the intact ion pair, and the HOMO and LUMO energy levels were obtained based on this configuration. Although the ionic additive is expected to dissociate in the electrolyte during battery operation, the calculations were intentionally conducted for the intact ion pair to illustrate the intrinsic frontier orbital energy levels and the spatial localization of the HOMO and LUMO electron densities. The HOMO, which is primarily associated with oxidation, was found to be localized on the anion, whereas the LUMO, which is primarily associated with reduction, was localized on the cation.

Figure 1c presents the non‐faradaic currents obtained in cyclic voltammetry experiments for electrolytes with different additive concentrations designed to verify the migration of the dissociated ions toward the electrode surfaces, with the corresponding cyclic voltammograms presented in Figure S1. It is worthwhile to note that the The measurements were conducted in both the low‐ and high‐potential regions, corresponding to the operation windows of the negative and positive electrodes, respectively. It was observed that the non‐faradaic current resulting from electrical double‐layer formation increased proportionally with the additive concentration. Because the non‐faradaic current originates from ion migration toward the electrode surface rather than from redox reactions, these results confirm the spontaneous migration of ionic species under an electrochemical field. In addition, the ionic conductivity was measured as a function of the additive concentration in the EC/EMC solvents without supporting salts (Figure S2). The results show that the ionic conductivity increases proportionally with increasing additive concentration, indicating that the designed additive is dissociated in the electrolyte and contributes effectively to ion transport within the solution.

Further, the electrochemical quartz crystal microbalance (EQCM) evaluation with additive‐added electrolyte was performed to validate the decoupled ionic migration. The marginal voltage step of 1.0 mV was applied to compare mass increase of electrode at high and low voltage exposure before additive decomposition. (Figure S3) While the increase of electrode mass reaches 0.30 ng cm^−2^ at ‐1.0 mV exposure, the 0.05 ng cm^−2^ mass increment was observed at +1.0 mV step application. These results imply that the heavier cationic species preferentially migrated toward the negative electrode surface, whereas the lighter anionic species migrated toward the positive electrode surface.

Figure 1d presents the results of the EQCM measurements that reveal the mass evolution during repeated potential scans. If an effective passivation film is formed during the initial cycle, the subsequent mass increase should be significantly suppressed relative to that of the first scan. However, for the background electrolyte, a continuous mass growth of approximately 70% per cycle was observed at a low potential during the second and third scans, indicating persistent interfacial decomposition. By contrast, in the presence of the ionic additive, the mass increase was reduced to ∼15% in the second scan and ∼9% in the third scan, demonstrating a substantially improved passivation capability at low potentials. A similar trend was observed at high potentials where the additive‐containing electrolyte effectively suppressed further oxidative decomposition after initial film formation, in contrast to the background electrolyte for which a continued mass increase was observed. These results confirmed the enhanced interfacial stabilization enabled by the ionic additives.

Figure 1e shows the ToF‐SIMS three‐dimensional reconstructed map used to verify that the designed functional moieties were directly incorporated into the resulting interphase. In the designed ionic species, the S–O moiety is present in both the cation and the anion. However, the number of S–O bonds in the cation is significantly lower than that in the anion. Therefore, the concentration of S–O species on the graphite electrode is expected to be lower than that in the surface film deposited on the positive electrode. In contrast, the C–N moiety is present exclusively in the cation and absent from the anion; therefore, the observed C–N species can only originate from the moiety in cation. To evaluate the composition of the resulting surface films, ToF‐SIMS measurements were performed. Typically, ToF‐SIMS analysis is ppb‐level sensitive technique, which is appropriate to investigate the detailed chemical composition of passivation films [44, 45, 46]. Thus, the ToF‐SIMS measurements distinguish the effects of decoupled interfacial modification from the additive introduction into the electrolyte.

The ToF‐SIMS analysis of the resulting interfacial films revealed distinct chemical compositions on the negative and positive electrodes. In the SEI formed on the negative electrode, a high concentration of C–N bonds and only a low concentration of S–O bonds were observed, consistent with the composition of the cation species. In contrast, no cation‐derived C–N bonds were observed in the surface film formed on the positive electrode, whereas only S–O bonds were detected. These results indicate that the positive‐electrode surface film was formed from the triflate anion, while the negative‐electrode SEI was primarily derived from the cation species.

Consequently, for the graphite negative electrode, nitrogen‐containing species originating from cations are clearly observed, indicating the formation of a nitrogen‐rich SEI. By contrast, the NCM811 positive electrode shows strong signals associated with SO_3_‐derived species from the anion, confirming the formation of a sulfur‐containing surface layer. These results directly demonstrate the selective interfacial film formation driven by directional ion migration.

The electrochemical performance characteristics of the 1.2 Ah pouch cells were examined to evaluate the effectiveness of the ionic additive (Figure 2). Figure 2a shows differential capacity (dQ/dV) plots obtained during the initial formation process with and without the additive, and linear sweep voltammetry (LSV) results for the electrolytes with and without additives are shown in Figure S4. Consistent with the frontier molecular orbital analysis, the LSV results indicate that cathodic and anodic decomposition of the ionic additive occurred prior to the decomposition of the background electrolyte. This behavior was also reflected in the dQ/dV profiles. In the additive‐free electrolyte, a dominant peak associated with EC decomposition and the subsequent passivation film formation was observed. By contrast, upon the introduction of the additive, a distinct peak arising from the predecomposition process appeared at approximately 2.35 V, corresponding to the decomposition of the additive, followed by a decrease in the EC decomposition intensity. This indicates that a stable interfacial film was formed prior to solvent decomposition, thereby modifying the interfacial reaction pathway.

**FIGURE 2:**
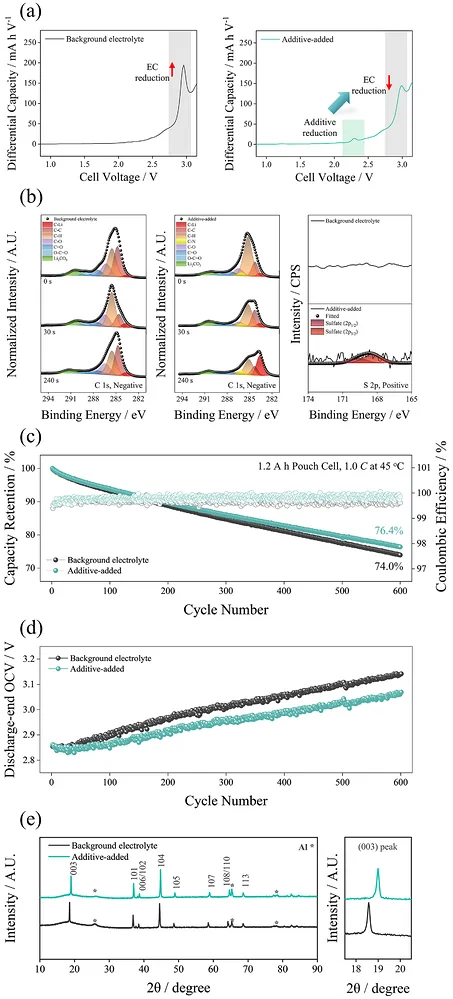
(a) Differential capacity plots, (b) *post‐mortem* C 1s and S 2p XPS spectra of graphite and NCM811 electrodes, respectively, (c) capacity retention during high‐temperature cycling, (d) cycle number‐dependent discharge‐end open‐circuit voltages obtained for graphite/NCM811 1.2 A h pouch cells with the background and additive‐added electrolytes, (e) XRD patterns of NCM811 electrodes after 600 cycles.

Figure 2b shows the results of X‐ray photoelectron spectroscopy (XPS) of the passivation layers formed on the graphite and NCM811 electrodes. As observed from the C 1s spectra, the additive‐added sample exhibits peaks corresponding to C–N bond [47, 48], confirming the incorporation of nitrogen‐containing compounds into the SEI film. The peak corresponding to the C–N bond in the N 1s spectrum is shown in Figure S5, indicating that the C–N‐based interface layer exists on the graphite electrode. Depth profiling at 30 and 240 s further revealed that the SEI formed in the additive‐added electrolyte was thinner than that formed in the background electrolyte, which is consistent with the suppressed EC decomposition during SEI formation. In addition, sulfate‐derived surface species were detected on the positive electrode surface [30, 39, 49], confirming the formation of a sulfur‐containing surface film. These results demonstrate the successful decoupled interfacial passivation on both electrodes. The etching time in XPS depth profiling depends on the chemical composition of the SEI; therefore, the absolute SEI thickness cannot be directly compared based solely on sputtering time. As complementary evidence, the Li–C signal originating from lithiated graphite was analyzed, where a higher Li–C intensity indicates a thinner residual SEI layer. The stronger Li–C signal observed for the additive‐containing electrode at the same sputtering time independently confirms that the additive suppresses SEI growth [50, 51, 52].

Figure 2c presents the results of the cycling performance evaluation of 1.2 A h graphite/NCM811 pouch cells operated at 45°C. It is observed that the ionic additive improves the overall coulombic efficiency over 600 cycles and leads to a 2.4% improvement in capacity retention. This enhanced performance was attributed to the formation of an effective initial passivation layer that suppressed continuous electrolyte decomposition. By contrast, the background electrolyte formed an insufficient protective layer, leading to sustained parasitic reactions and progressive electrolyte consumption during cycling. The cycle performance of the cells surpassed the performance achieved with the previously reported additive molecules (Table S1).

Continuous electrolyte decomposition consumes both the electrolyte and lithium, resulting in a gradual reduction in the available cyclable Li ions which in turn leads to a progressive increase in the discharge‐end open‐circuit voltage (OCV) during cycling. Figure 2d shows the cycle‐dependent discharge‐end OCV evolution, and it is observed that the background electrolyte‐injected cell exhibited a pronounced increase in the discharge‐end OCV, whereas the additive‐added cell showed a much more moderate increase, indicating effective suppression of lithium inventory loss. The suppression of the lithium inventory loss by the additive was further verified by calculations of the irreversible capacity during cycling. The cumulative irreversible capacity is plotted in Figure S6 and the results clearly show that the system with the additive‐added electrolyte exhibits a substantially lower accumulative irreversible capacity than the system with the background electrolyte. This further indicates that parasitic side reactions and continuous lithium consumption were significantly suppressed in the presence of the ionic additive, leading to improved cell balance stability over prolonged cycling.

To directly confirm lithium retention, *ex‐situ* X‐ray diffraction (XRD) analysis was performed on the positive electrode after cycling. The (0 0 3) diffraction peak shifted to higher angles in the additive‐added sample compared to the sample with the background electrolyte [1, 7, 53, 54], indicating a reduced interlayer spacing associated with a higher lithium content in the layered structure. The representative (0 0 3) peak was compared because this peak position directly reflects the lithium content in layered oxide materials [55, 56]. This confirms that the ionic additive effectively suppressed parasitic lithium consumption during cycling, leading to improved lithium inventory retention.

Interfacial degradation and resistance evolution after long‐term cycling were examined (Figure 3). Figure 3a shows the XPS results obtained for the cycled graphite negative electrodes for systems with the background and the additive‐added electrolytes. As observed from the C 1s spectra, the background electrolyte exhibited a substantial increase in the C‐O and O‐C = O species [5, 6, 7, 57] that are typically associated with EC‐derived decomposition products, indicating extensive growth of the SEI film due to insufficient initial passivation. By contrast, the additive‐added electrolyte suppressed the formation of these EC‐derived species, which is consistent with the effective protection of the electrode surface by the nitrogen‐containing SEI. For the F 1s spectra, the additive‐added sample also shows a reduced intensity of the LiPF_6_ decomposition products such as Li*_x_* PO*_y_* F*_z_* and Li*_x_* PF*_y_* [58, 59, 60], indicating effective mitigation of continuous salt decomposition.

**FIGURE 3:**
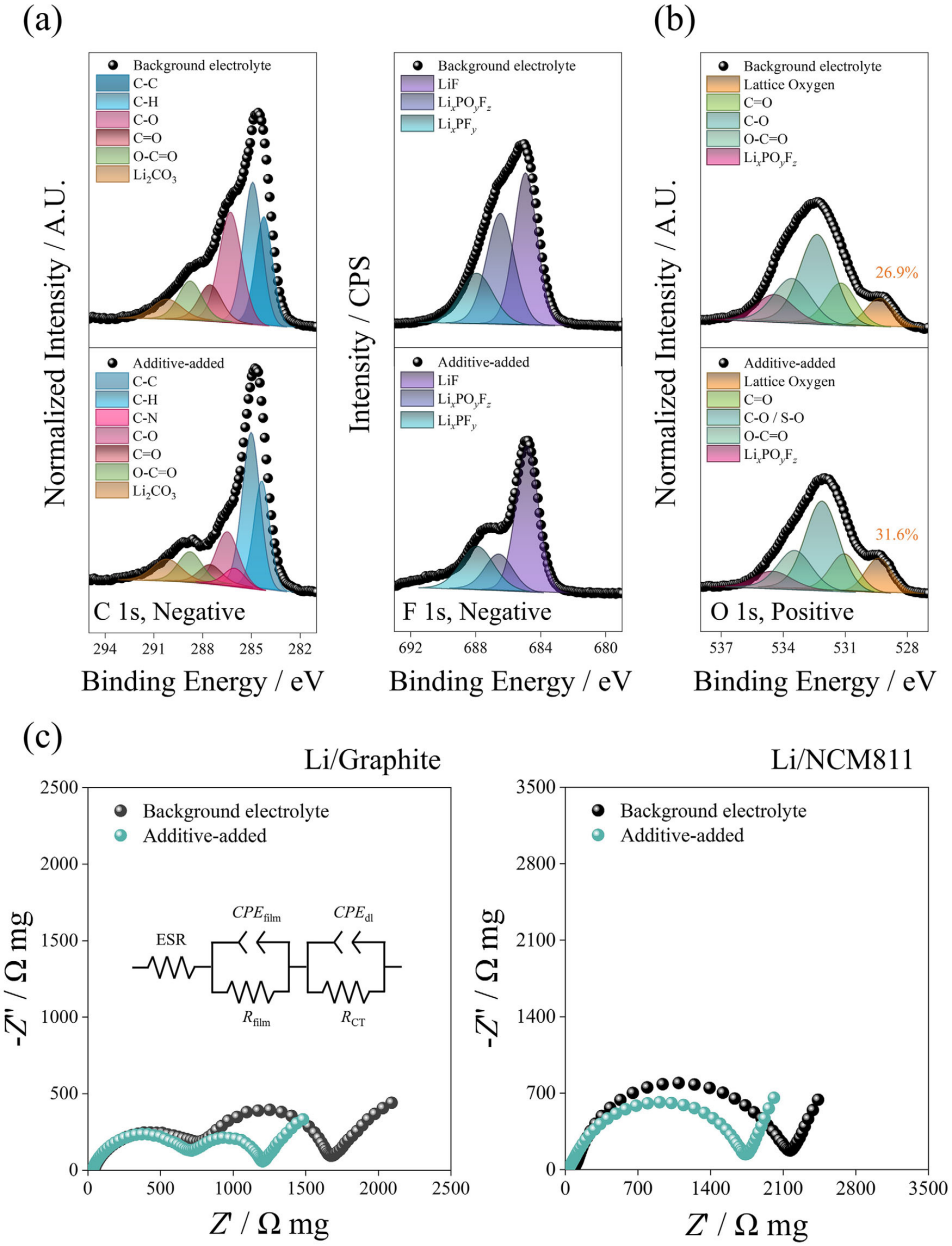
*Post‐mortem* (a) C 1s and F 1s XPS spectra obtained from cycled negative electrodes, (b) O 1s XPS spectra from positive electrodes, (c) Nyquists plots recorded from reassembled Li/graphite and Li/NCM811 half‐cells after cycling.

Figure 3b shows the XPS spectra of the cycled NCM811 positive electrodes. For the additive‐added electrolyte, the accumulation of the C‐O and C = O species on the electrode surface decreased significantly compared to that for the background electrolyte. Consequently, the relative contribution of the lattice‐oxygen‐related signals increased, indicating decreased coverage by the electrolyte decomposition products and improved interfacial stability.

Figure 3c shows the Nyquist plots obtained from the Li/graphite and Li/NCM811 half‐cell configurations assembled after cycling which are used for the deconvolution of the resistance contributions for each electrode. Both electrodes cycled in the presence of the additive demonstrated suppressed polarization growth after cycling compared to the cycled electrodes with the background electrolyte, implying that the failure of the graphite and NCM electrodes was mitigated by the reinforced interfaces (Figure S7). For the Li/graphite cells, the additive‐added system exhibited a suppressed increase in the electrochemical series resistance (*ESR*), which was attributed to suppressed SEI growth and the subsequent limited accumulation of resistive interphase films. In addition, both the SEI resistance and charge‐transfer resistance (*R*_CT_) were significantly lower than those of the system with the background electrolyte. A similar trend is observed for the positive electrode as the additive‐added electrolyte suppressed the increase in *ESR* and markedly reduced the growth of *R*_CT_. These results confirm that the ionic additive effectively suppresses interfacial side reactions, thereby mitigating resistance growth in both electrodes after prolonged cycling.

The morphological changes of the negative and positive electrodes after long‐term cycling were examined by scanning electron microscopy (SEM) and X‐ray microscopy (XRM) (Figure 4). Figure 4a,b present the cross‐sectional and top‐view SEM images of the graphite negative electrodes after cycling. Consistent with the *post‐mortem* XPS analysis, the cell with the background electrolyte exhibited insufficient suppression of electrolyte decomposition during cycling, leading to significant interphase resistance growth and severe pore clogging within the electrode structure. By contrast, in the additive‐added electrolyte, the growth of the SEI and the associated clogging of the electrode pores were substantially mitigated, resulting in a more intact porous architecture after cycling. This difference in the pore preservation directly influences the evolution of electrode impedance. In the case of the background electrolyte, severe impedance build‐up promoted lithium plating on the negative electrode surface, whereas the additive‐incorporating cell maintained a clean and uniform surface without evident Li plating. These results indicate that suppression of interfacial degradation plays a crucial role in improving both cycleability and cell safety. Figure 4c shows XRM images of the cycled graphite electrode with and without additives. The XRM map represents the volume of the pores merged along the z axis and thus intuitively represents the degree of preservation of the pore structure inside the electrode. While the background electrolyte showed a decreased pore volume in the graphite electrode, the additive‐incorporating electrode demonstrated a well‐preserved electrode architecture. This implies that further deposition of the SEI film on the graphite electrode was effectively suppressed by the ionic additive. Furthermore, because the introduction of ionic additives effectively suppressed electrode pore clogging, smooth ion transport within the electrode was maintained, further verifying the suppression of Li plating on the electrode surface. Figure 4d,e show the cross‐sectional and top‐view SEM images of the positive electrodes after cycling. For the system with the background electrolyte, significantly higher levels of surface byproduct accumulation were observed than in the additive‐incorporating system, indicating pronounced oxidative electrolyte decomposition at the electrode surface. Correspondingly, the cross‐sectional images reveal severe intergranular cracking within the secondary particles of NCM811 for the background‐electrolyte sample, whereas the additive‐incorporating electrolyte effectively suppressed the surface degradation and structural cracking. These results suggest that impedance growth in the positive electrode is not only driven by surface film thickening due to electrolyte oxidation but is also strongly associated with the mechanical degradation of secondary particles induced by interfacial instability.

**FIGURE 4:**
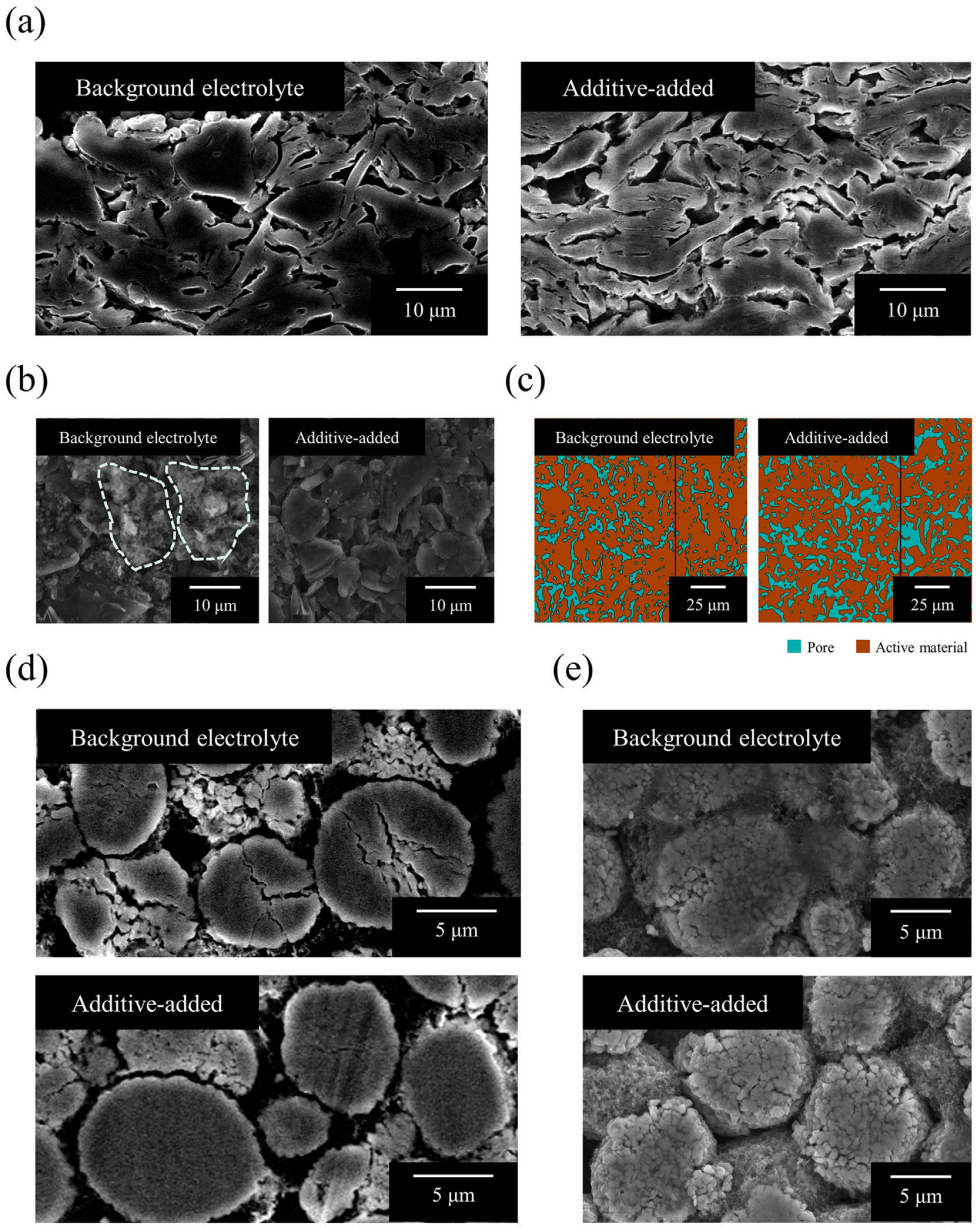
(a) Cross‐sectional and (b) top‐view SEM images of cycled graphite electrodes, (c) XRM images of cycled graphite electrodes and (d) cross‐sectional and (e) top‐view SEM images of cycled NCM811 electrodes with background and additive electrode.

The fundamental working principle of ionic additives is based on the effect of directional ion migration on failure mitigation in lithium‐ion batteries as schematically illustrated in Figure 5. During the SEI formation process, the electrochemical potential gradient induces spontaneous migration of dissociated ionic species within the electrolyte. Specifically, in response to evolving electrode potentials, cations migrate toward the negative electrode while anions migrate toward the positive electrode. By incorporating film‐forming functionalities into each ionic species, this directional transport behavior enabled spatially selective interfacial film formation on both electrodes. The cation‐derived species preferentially decompose at the negative electrode, forming a nitrogen‐rich SEI that effectively stabilizes the electrode surface and suppresses continuous electrolyte reduction. Concurrently, the anion‐derived species migrate toward the positive electrode and form a sulfur‐containing surface film that mitigates oxidative electrolyte decomposition and structural degradation. In the absence of an ionic additive, continuous interfacial side reactions lead to progressive resistance growth at the negative electrode which in turn promotes Li plating because of the increased polarization of graphite from pore clogging. This parasitic Li deposition accelerates cell degradation and safety deterioration. By contrast, the decoupled interfacial passivation strategy simultaneously suppresses key failure pathways in lithium‐ion batteries, including electrolyte decomposition, continuous loss of active lithium inventory, resistance growth, and lithium plating. Consequently, the additive design enabling directional ion migration provides a unified mechanism for stabilizing both electrodes using a single additive molecule, thereby enhancing the overall cycleability and mitigating long‐term electrochemical and morphological degradation.

**FIGURE 5:**
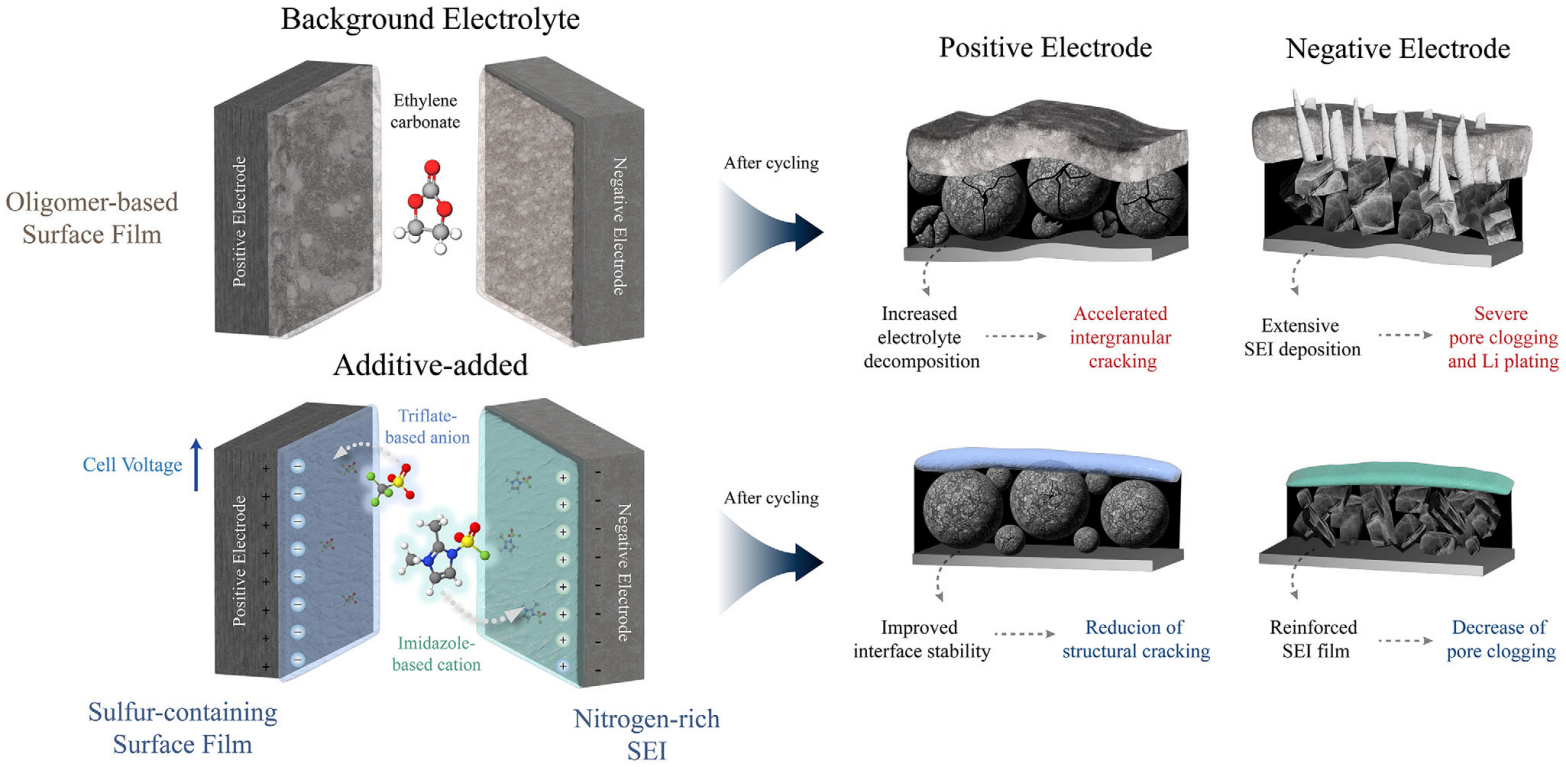
Schematic illustration of directional ion migration effect on failure mitigation in lithium‐ion batteries.

## 3 Conclusion

An individually functionalized ionic additive was designed to enable directional‐ion migration‐driven decoupling of interfacial passivation in lithium‐ion batteries. This strategy exploits the intrinsic migration behavior of dissociated cations and anions under electrochemical polarization to achieve spatially selective formation of protective films on both electrodes using a single additive.

Fundamental analyses confirmed that the ionic additive underwent preferential decomposition before the background electrolyte, enabling early stage passivation film formation that effectively modified the subsequent parasitic electrolyte decomposition. Electrochemical and spectroscopic results demonstrated that cation‐derived nitrogen‐containing species formed a stable SEI film on graphite while anion‐derived sulfonate species formed a robust film on the high‐Ni positive electrode. This decoupled interfacial chemistry suppresses continuous electrolyte decomposition, salt degradation, and excessive interphase growth, thereby reducing irreversible lithium consumption and improving interfacial stability.

Full‐cell evaluations further confirmed that the ionic additive significantly enhanced the electrochemical performance of the graphite/NCM811 pouch cells, delivering improved capacity retention, higher Coulombic efficiency, and suppressed impedance growth during extended cycling at elevated temperatures. In addition, the reduced rise of the discharge‐end OCV and lower accumulative irreversible capacity indicate effective mitigation of active lithium loss. *Post‐mortem* investigations revealed that the additive suppressed morphological degradation, including pore clogging and Li plating on the negative electrode and intergranular cracking of NCM secondary particles, thereby enhancing both performance stability and safety.

Thus, this work demonstrates that directional ion migration can be utilized as a design principle for electrolyte additives, enabling simultaneous and selective stabilization of both electrode interfaces. This strategy provides a general and effective approach for interfacial engineering in high‐energy‐density LIBs, offering a pathway toward improved long‐term cycleability and failure mitigation in practical LIB systems.

## Conflicts of Interest

The authors declare no conflicts of interest.

## Supporting information




**Supporting File**: smll74978‐sup‐0001‐SuppMat.docx.

## Data Availability

The data that support the findings of this study are available from the corresponding author upon reasonable request.
