# Supplementary material for: Decoupled Interface Passivation Enabled by Directional Ion Migration in Lithium‐Ion Batteries
Source: Small. 2026 Aug 2;22(56):e74978. doi: 10.1002/smll.74978 (PMC13653351; doi:10.1002/smll.74978)
Supplement: Supplementary file 1 [file SMLL-22-e74978-s001.docx]

Supporting Information

**Decoupled Interface Passivation Enabled by Directional Ion Migration in Lithium-ion Batteries**

*Juhwi Park ^a^, Jooeun Byun ^b^, Chae Rim Lee ^b^, and Hyun-seung Kim ^b,c,d*^*

**Experimental**

***Electrolyte formulation***

Ionic additive (1-(fluorosulfonyl)-2,3-dimethyl-1*H*-imidazol-3-ium trifluoromethanesulfonate) (Sigma-Aldrich) was added to 1.0 *M* LiPF_6_ EC/EMC (3:7=*v*/*v*, Dongwha Electrolyte) at 0.5 *wt*.% concentration. This mixture was shaken in an Ar-filled glove box for 24 h. The ionic conductivities for each additive concentration without LiPF_6_ were measured at 20.9 ^o^C using a conductivity meter (Oakton CON 6+).

***Electrode fabrication and pouch cell evaluation***

The positive electrode was prepared by dispersing LiNi_0.8_Co_0.1_Mn_0.1_O_2_ (NCM, BTR), conductive carbon (Super P, TIMCAL), and poly(vinylidene fluoride) (PVDF, SOLVAY) in N-methyl-2-pyrrolidone (NMP, Sigma-Aldrich) in a weight ratio of 96:2:2 to form a uniform slurry. This slurry was coated onto a 15 μm aluminum current collector at an area capacity of 3.5 mA h cm⁻² and a slurry density of 3.2 g cm⁻³. The coated electrodes were dried at 120 °C overnight prior to cell assembly. The negative electrode was prepared by casting a deionized-water-based slurry of artificial graphite (BTR), conductive carbon (Super P, TIMCAL), and styrene-butadiene rubber-carboxymethyl cellulose (SBR-CMC, Sigma Aldrich) in a weight ratio of 95:2.5:2.5 onto a 20 μm copper current collector.

Pouch cells with a nominal capacity of 1.2 A h were assembled by stacking a positive electrode, a polyethylene separator, and a negative electrode. The negative-to-positive electrode capacity ratio (N/P ratio) was 1.08. The electrolyte (3 g, E/C ratio of 2.5) either with or without the additive was injected and then the cells were degassed (Topre) and allowed to rest at room temperature for 24 h. Subsequently, the cells were pressurized to 1.3 N·m to ensure uniform electrode contact.

Cell formation (TOYO Japan) was carried out under a constant current (CC) charge of 0.1 *C* to 4.2 V, followed by a constant voltage (CV) hold until the current decreased to 0.05 *C*, and a CC discharge at 0.1 *C* to 2.5 V. Following formation, cycling performance was evaluated at 45 °C using a CC–CV charge of 1.0 *C* (0.05 *C* cut-off) and a CC discharge of 1.0 *C* within a voltage range of 2.5–4.2 V.

***Calculation Details***

Theoretical calculations to estimate the HOMO and LUMO energy levels of the electrolyte and additive were carried out with ORCA 4.1.1 using the B3LYP functional and a triple-zeta valence polarization (TZVP) basis set.

***Cyclic voltammetry (CV) analysis***

CV tests were performed using a BioLogic potentiostat with Li/Cu and Li/Al half-cells to verify the currents resulting from the migration of the additive molecules. Cu and Al foils were used as working electrodes, and Li metal (mixed metal) was used as the counter and reference electrodes. Electrolytes at various additive concentrations without LiPF_6_ salts were prepared and used to investigate the non-faradaic currents at a scan rate of 1 mV/s. The scans were performed from the open-circuit voltage (OCV) to 1.5 V (*vs*. Li/Li^+^) and from OCV to 3.3 V (*vs*. Li/Li^+^), respectively. Experiments were conducted in a dry room.

***Linear Sweep Voltammetry (LSV) analysis***

To investigate the electrochemical decomposition behavior of the additive-based electrolytes, LSV tests were performed using Li/Cu and Li/Al half-cells in a dry room. Li metal was used for the counter and reference electrodes, and Cu and Al foils were used as the working electrodes to examine the reduction and oxidation currents, respectively. The voltage was scanned from OCV to 0 V (*vs*. Li/Li^+^) and from OCV to 5.0 V (*vs*. Li/Li^+^) at a scan rate of 0.1 mV s^-1^.

***Electrochemical quartz crystal microbalance (EQCM) analysis***

A Seiko EG&G system was used with a PEEK-based cell in a dry room to measure the changes in the electrode mass. A Pt-sputtered quartz crystal electrode was used as the working electrode, and Li metal was used for the counter and reference electrodes. To evaluate the mass change after low voltage exposure, a voltage sweep experiment was performed at a scan rate of 5.0 mV s^-1^. The voltage was scanned from OCV to 0.3 V (*vs*. Li/Li^+^) for the negative potential range and from OCV to 4.2 V (*vs*. Li/Li^+^) for the positive potential range.

***Spectroscopic and Morphological Analysis***

Time-of-flight secondary ion mass spectrometry (ToF-SIMS) (ION-ToF) was used to investigate the surface film components of the electrodes after SEI formation. X-ray photoelectron spectroscopy (XPS, Thermo Fisher Scientific) was used to confirm the chemical bonding states of the surface elemental species. The cells were then disassembled in a glovebox filled with Ar. To remove the residual solvent and Li salt, the electrodes were rinsed with EMC (Dongwha Electrolyte) and dried under vacuum for 12 h.

***Post-mortem structural analysis***

X-ray diffraction (XRD, Rigaku D/Max-2500V) was conducted to determine the structure of cycled NCM811 electrodes using Cu Kα radiation. Diffraction patterns were collected in the 2θ range of 10–90^o^ at a step size of 0.02^o^ min^−1^. To eliminate instrumental peak position deviations, all diffraction patterns were corrected relative to the Al current collector peak. The corrected diffraction patterns were analyzed using the FullProf program, and the relative lithiation state of the cycled anode after full discharge was evaluated to compare the (0 0 3) peak positions.

***Electrochemical impedance spectroscopy (EIS)***

Electrochemical impedance spectroscopy (EIS) measurements were performed in the frequency range from 3 MHz to 10 mHz at an amplitude of 5.0 mV using a Biologic potentiostat. The cycled pouch cells were then disassembled in a fully discharged state after 600 cycles in a dry room. The positive and negative electrodes were rinsed with EMC and vacuum-dried for 12 h. A 2032-type coin cell was fabricated using positive and negative electrodes in the state of charge (SOC) 0 as the working electrode, a polyethylene separator, and lithium metal foil as the counter and reference electrodes. 1.0 *M* LiPF_6_ EC/EMC (3:7=v/v) electrolyte (60 μL) was injected into the coin cell. The EIS of the fabricated cell was performed at an SOC of 50%.

**Fig. S1** Cyclic voltammetry for systems with different additive concentrations

**Fig. S2** Ionic conductivity as a function of additive concentration

**Figure S3.** Mass increase after 1.0 mV potential step application at additive-added electrolyte

**Figure S4.** LSV profiles of Li/Cu and Li/Al systems with the background and additive-incorporating electrolytes

**Table S1.** Comparison of the electrolyte additive with previously reported additives

| **Electrolyte** | **Additives** | **Cell type** | **Cut-off** | **C-rate** | **Performance**  **(cycle/retention)** | **Temperature** | **Ref.** |
| --- | --- | --- | --- | --- | --- | --- | --- |
| 1 *M* LiPF_6_ in EC:DMC:PC  =1:3:1 (v/v/v) | Fluoroethylene Carbonate (FEC) 2 wt%  Vinylene Carbonate (VC) 2 wt%  1,3-Propane Sultone (PS) 2 wt% | Three-electrode  T-type Swagelok cells  Graphite/  NCM532 | 4.5-3.0 V | 1.0 *C* | 120 cycles  w/o additive: 84.7%  88.8%  90.7%  88.1% | 20±1 °C | Adv. Mater. Interfaces 7 (2020) 1901500 |
| 1 *M* LiPF_6_ in  EC:DMC  =3:7 (v/v) | Lithium difluorophosphate (LiDFP)  1 wt% | Coin cell  Graphite/LMR | 1.9-4.5 V  1.9-4.7 V | 1.0 *C* | 102 cycles  86%  47% | 20 °C | Energy Environ. Mater. 8(2) (2025) e12835 |
| 1 *M* LiPF_6_ in  EC:DMC  =1:1 (wt/wt) | 4,4-diaminodiphenylsulfone (DADP)  0.2 wt%  + dimethyl methyl phosphonate (DMMP)  5 wt% | Cylindrical 18,650 cell  Graphite/  NCM523 | 3.0-4.2 V | 0.5 *C* | 650 cycles  52.67% | Ambient temperature | Mater. Today Sustain. 28 (2024) 101004 |
| 1.15 *M* LiPF_6_  EC:EMC:DEC  =2:5:3 (v/v/v) | Ethyl 4,4,4-trifluorobutyrate (ETFB)  1 wt% | Coin cell  Graphite/  NCM523 | 3.0-4.35 V | 0.5 *C* | 300 cycles  84.8% | 45 °C | J. Power Sources 396 (2018)  276-287 |
| 1.0 *M* LiPF_6_ in  EC:EMC  =3:7 (v/v) | Lithium tetrafluoro(fluoromalonato)phosphate (LFMP)  1 wt% | Coin cell  Graphite/  NCM811 | 3.0-4.3 V | 1.0 *C* | 250 cycles  52% | 60 °C | Energy Storage Mater. 50 (2022)  75-85 |
| 1 *M* LiPF_6_  EC:EMC:DEC  =3:4:3  (v/v/v) | 3-(trimethylsilyl)-  2-oxazolidinone (TMS-ON)  0.5 wt% | Coin cell  Graphite/  LiN_0.7_Co_0.15_M_0.15_O_2_ | 3.0-4.35 V | 0.5 *C* | 400 cycles  80.4% | 45 °C | Adv. Energy Mater. 10(15) (2020) 2000012 |
| 1 *M* LiPF_6_ in  EC:EMC  =3:7 (v/v) | 1-(Fluorosulfonyl)-2,3-dimethyl-1*H*-imidazol-3-ium trifluoromethanesulfonate  0.5 wt% | 1.2 A h pouch cell  Graphite/  NCM811 | 2.5-4.2 V | 1.0 *C* | 600 cycles  76.4% | 45 °C | This work |

**Figure S5.** *post-mortem* N 1s XPS spectra of graphite electrode with additive-added electrolyte

**Figure S6.** Calculated accumulated irreversible capacity of the graphite/NCM811 cells with background and additive-incorporating electrolytes

**Figure S7.** Nyquist plots for Li/graphite and Li/NCM811 cells with the background and additive-incorporating electrolytes
